# Moxidectin: heartworm disease prevention in dogs in the face of emerging macrocyclic lactone resistance

**DOI:** 10.1186/s13071-021-05104-7

**Published:** 2022-03-11

**Authors:** Molly D. Savadelis, Tom L. McTier, Kristina Kryda, Steven J. Maeder, Debra J. Woods

**Affiliations:** Zoetis, Kalamazoo, MI USA

**Keywords:** *Dirofilaria immitis*, Heartworm, Macrocyclic lactone, Moxidectin, Prevention, Resistance

## Abstract

Heartworm (*Dirofilaria immitis*) disease continues to increase and spread, remaining one of the most important and pathogenic parasitic diseases of dogs, despite the regular use of macrocyclic lactones (MLs) in preventive products. Dogs harboring strains of *D. immitis* resistant to MLs, the only drug class available for heartworm prevention in the United States, have been documented and proven. As no new products are available utilizing a novel drug class for the prevention of this disease, the only options for combating ML resistance include increasing the dose and/or changing the dosage regime of current MLs, or by optimizing the formulations of MLs currently available. Moxidectin provides a unique opportunity for optimization of the dose and formulation, which may provide improved efficacy against ML-resistant strains. Currently there are oral, topical, and injectable moxidectin products approved for heartworm prevention in the USA. Two new products (ProHeart^®^ 12 and Simparica Trio^®^), available in many countries around the world including the USA, take advantage of the unique attributes of moxidectin for providing robust heartworm prevention against the strains of heartworm to which most dogs in the USA will likely be exposed. Both products have demonstrated 100% preventive efficacy in laboratory studies against recently collected field strains of heartworm, and also in large field studies, where the majority of dogs were living in the southern USA in areas where ML resistance has been confirmed to occur, therefore under elevated heartworm challenge. Based on the data summarized here, these products offer important advances in heartworm prevention and provide additional options for veterinarians and pet owners to protect their dogs from developing heartworm disease.

**Graphical Abstract:**

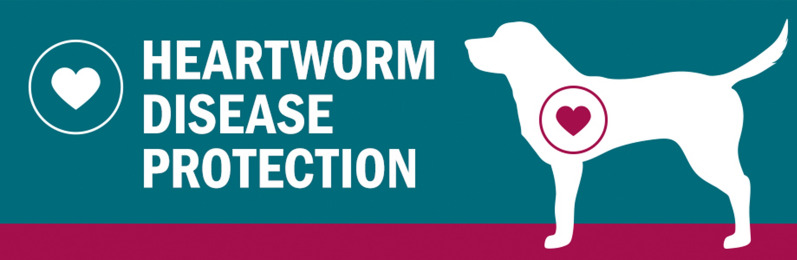

## Background

The causative agent of heartworm disease, *Dirofilaria immitis,* infects many mammals including dogs, cats, and ferrets [1, 2]. This parasitic filarial disease is characterized by the presence of sexually mature dimorphic adults in the pulmonary arteries that produce microfilariae (MF) circulating in the bloodstream [3]. Female mosquitoes ingest circulating MF during a blood meal from animals harboring patent infections, with ingested MF molting to the infective third-stage larvae (L3) within 10–14 days post-blood meal. Thereafter, during subsequent blood meals, the L3s are deposited on the skin of the host and enter the subcutaneous tissues through the bite wound. The L3s molt to the fourth-stage larvae (L4) 3–5 days post-infection in the host, reaching the heart and lungs as early as 70 days post-infection as juvenile adult worms (L5) [1].

The presence of adult worms in the pulmonary arteries causes vascular damage such as thickening of the arteries and hypertrophy of the smooth muscle cells [4, 5]. Vascular changes, including separation of endothelial junctions, loss of endothelial cells, and the adherence of leukocytes due to the presence of adult worms, can be detected as soon as 4 days post-surgical transplantation of adults in heartworm-naïve dogs [6]. This damage appears to be chronic and cumulative, with increasing numbers of worms and duration of infection causing more severe pathological changes. Pathological lesions in the vasculature due to the presence of adult worms is caused by endothelial swelling, precipitating the accumulation of platelet-derived growth factor (PDGF) [7]. This accumulation of PDGF stimulates the migration of smooth muscle cells into the tunica media, causing the rough velvety appearance observed in the pulmonary arteries in heartworm disease [8].

The vascular changes and pathology associated with heartworm disease include right heart enlargement, main and lobar pulmonary artery enlargement and tortuous vasculature, with resulting congestive heart failure, hypertension, and the potential development of caval syndrome over time [4, 9]. While melarsomine dihydrochloride is approved for the treatment of adult heartworms in canine heartworm disease, there are no approved therapeutics for the treatment of adult heartworms in cats. With limited adulticidal treatments available, the severity of damage associated with this disease, and the potential complications associated with its treatment, the prevention of heartworm disease development is crucial [10].

Heartworm disease has been successfully prevented by the proper administration of chemoprophylactic medications in dogs and cats. Current products on the market prevent heartworm disease by targeting the L3 and L4 stages, preventing the development of larval heartworms into adults and subsequent migration of adults to the heart and pulmonary arteries through the use of various macrocyclic lactone (ML) formulations. MLs have been successfully utilized for the prevention of heartworm disease for over 30 years [11–19]. Within this class of molecules are the avermectin and milbemycin subfamilies, of which ivermectin, eprinomectin, abamectin, selamectin, and doramectin are classified under the avermectin subfamily, and milbemycin oxime and moxidectin under the milbemycin subfamily [13]. These molecules are available in various commercial presentations approved by the Food and Drug Administration (FDA) Center for Veterinary Medicine (CVM) as oral, topical, and injectable products.

While MLs have been successfully utilized for the prevention of heartworm disease, and for the treatment and control of numerous other parasitic infections for decades, the exact mechanism of action of this drug class in the prevention of heartworm disease is unclear. MLs have been shown to bind to glutamate-gated chloride ion channels precipitating hyperpolarization, paralysis, and death of heartworms [20–22]. In mammals, MLs bind to gamma-aminobutyric acid type A-gated chloride (GABA_A_) channels and glycine receptors located in intestinal epithelial cells, central nervous system neurons, brain capillary endothelial cells, renal epithelial cells, placenta, and testes [23]. GABA_A_ channels are protected by ATP-dependent transmembrane efflux pumps, P-glycoproteins (P-gp), which actively pump xenobiotics out of the cell. ABCB1 mutations, such as multidrug resistance-1-mutants (MDR-1) result in improper development of P-gps, featuring a premature stop codon in the ABCB1 gene. Homozygous MDR1-mutant dogs have an increased sensitivity to MLs due to the abnormally high accumulation of drug by the nonfunctional P-gp formation. Additionally, MDR1-mutant dogs are susceptible to increased neurological side effects due to the increased accumulation of drug in the central nervous system [23]. However, all FDA CVM-approved heartworm preventive products are safe to administer to wild-type and MDR1-mutant dogs at the manufacturer’s recommended dose.

Despite the high potency of the MLs against *D. immitis* L3 and L4 stages in vivo, the concentrations of MLs necessary in vitro to kill these stages do not correlate with the in vivo potency, making pharmacokinetic/pharmacodynamic relationships unpredictable. This drug class has been observed to bind at the excretory-secretory (ES) pore of *Brugia malayi* MF, a filarid closely related to *D. immitis,* and reduce the concentration of ES products post-treatment [24, 25]. These ES products are theorized to suppress the host immune response and bias the host toward an anti-inflammatory Th2 response in order to evade the host immune system. Therefore, it is hypothesized that the host immune response plays an integral role in the killing of these larvae post-ML treatment [26]. It is possible that these same mechanisms are also involved in the activity of MLs against *D. immitis*.

### Macrocyclic lactone resistance in heartworm disease

Despite the high efficacy of heartworm preventive products, the prevalence of reported heartworm-positive cases increased by 21.7% between 2013 and 2016 [27], and resistance of *D. immitis* to MLs has been confirmed against multiple strains of heartworms in the USA [27–33]. Without any new products on the market for heartworm prevention utilizing a novel mechanism of action, the management of ML resistance may currently only be addressed by optimizing the available MLs. This can be achieved by increasing the dose of the ML, the frequency of dosing, and/or improving the bioavailability of the drugs through optimized formulations. Among the MLs, moxidectin has some unique attributes such as high potency, a long half-life and other favorable pharmacokinetic parameters, a wide therapeutic index, and versatility in formulation that may allow the development of new products that could aid in overcoming drug resistance [34].

To date, confirmed cases of ML resistance have been primarily concentrated in the Lower Mississippi River Valley (LMRV) region, but with the continual movement of dogs around the USA and the availability of competent mosquito vectors across the USA, the spread of ML-resistant heartworms is inevitable [35]. Dogs rescued by shelter organizations harboring heartworm infections are regularly transported across state lines, with many of these dogs coming from the LMRV, where ML resistance has been confirmed [36, 37]. This further increases the likelihood of the spread of ML resistance to other areas of the USA. The use of products with proven preventive efficacy against ML-resistant heartworm infections may help slow the spread of these infections and even prevent the establishment of new resistant *D. immitis* subpopulations.

### Moxidectin pharmacology

MLs are 16-member rings fused with both benzofuran and spiroketal functions that target glutamate-gated chloride ion channels in invertebrates, including filarial nematodes. Of all the ML molecules, moxidectin has the most unique characteristics that enable optimal product profiles. Most notably, moxidectin is the most lipophilic and potent of the MLs [34, 38, 39].

With lipophilicity of log*P*_MOX_ = 6 and log*P*_IVM_ = 4.8, moxidectin has a higher tissue distribution compared to ivermectin, leading to a lower rate of clearance [38]. As heartworm L3s and L4s migrate through host tissue during their pathway to the pulmonary arteries, higher tissue distribution and longer elimination half-life may play a role in the increased potency of moxidectin as compared to other MLs [2, 34]. Moxidectin was shown to be 100% effective when dosed 60 days post-infection at only 0.5 µg/kg compared to the lowest efficacious doses of 6 µg/kg and 500 µg/kg for ivermectin and milbemycin oxime, respectively, when administered 30 days post-infection against a known susceptible heartworm strain (Table 1) [15, 40–42].

**Table 1:** Preventive efficacy of orally administered moxidectin, ivermectin, and milbemycin oxime against ML-susceptible *Dirofilaria immitis* at various dosages and treatment times post-inoculation

Macrocyclic lactone (FDA-approved dose)	Dosage (µg/kg)	Treatment time post-inoculation (months)^a^	Preventive efficacy (%)	References
Moxidectin	0.5	2	100	[15]
(3 µg/kg)	0.625	2	100	[15]
	1.0	3	47.8	[15]
	1.25	1	100	[15]
	1.25	2	100	[15]
	3.0	1	100	[15]
	3.0	3	64.2	[15]
Ivermectin	1	1	53.2	[42]
(6 µg/kg)	2	1	83.3; 97.2	[41, 42]
	2	1.5	63.8	[42]
	3.3	1	98.1	[42]
	6	1	100	[41]
	6	1.5	100	[41]
Milbemycin oxime	500	1	100	[40]
(500 µg/kg)	500	1.5	100	[40]
	500	2	93.9	[40]

^a^Dogs were inoculated with 30–50 third-stage larvae utilizing a macrocyclic lactone-susceptible strain

In addition to the increased lipophilicity and potency, moxidectin displays a lower affinity to GABA_A_ channels leading to an increased safety profile for this molecule in MDR-1ab(−/−) P-gp deficient mice [43]. ML toxicity is characterized by the oversaturation of drug binding to P-gps and the accumulation of drug in the brain. P-gps actively transport drugs across membranes, protecting such areas as the blood–brain-barrier. In dogs homozygous for the MDR-1 mutation, moxidectin has demonstrated improved safety compared to ivermectin and doramectin, with only mild neurotoxicity when administered at ≥ 400–1000 µg/kg, while ivermectin and doramectin induce severe neurotoxicity when administered at 200–600 µg/kg [23, 44–47].

Studies characterizing the binding affinity of ivermectin and moxidectin have indicated that while moxidectin and ivermectin bind to glutamate-gated chloride ion channels, moxidectin may interact in a different manner than ivermectin due to structural differences such as the lack of a disaccharide moiety on C-13 of the macrocycle in moxidectin, an olefinic side chain on C-25, and a methoxime moiety on C-23 [34, 38]. These differences between moxidectin and ivermectin in sites of binding to nematode glutamate-gated chloride ion channels may be a contributing factor in the slower development of drug resistance to moxidectin. The mode of action, structural differences, and pharmacodynamics of MLs, including moxidectin, have been reviewed previously [34, 38].

### Moxidectin formulations available for heartworm prevention

Due to the versatility of moxidectin, a number of formulations are available for heartworm prevention. These include products for oral, topical, and injectable administration.

### Oral moxidectin

Initially, moxidectin was developed as a monthly oral dose product administered at 3 µg/kg labeled for heartworm prevention only. However, this product was marketed in the USA only for a short period of time but remains on the market in Latin America and in some areas of Southeast Asia. More recently, moxidectin has been formulated with other active pharmaceutical ingredients (APIs), sarolaner and pyrantel pamoate, for oral delivery as Simparica Trio^®^ (Zoetis, NJ, USA). This combination of APIs provides additional coverage against multiple ecto- and endoparasites. The Simparica Trio formulation (24 µg/kg moxidectin/1.2 mg/kg sarolaner/5 mg/kg pyrantel) provides a convenient, easy-to-administer option for pet owners, as well as eliminating concerns of potential human and environmental exposure, as is the case with topical products.

Evaluations of the preventive efficacy of a number of MLs, at various oral dosages and treatment times post-experimental inoculation, were performed for the determination of minimum efficacious dosages for heartworm prevention (Table 1). Ivermectin administered 30 days post-inoculation was not 100% efficacious in the prevention of heartworm disease at ≤ 3.3 µg/kg, with decreasing efficacy when administered 45 days post-inoculation compared to the 30-day post-inoculation efficacy [41, 42]. When administered at 30 or 45 days post-inoculation, 6 µg/kg ivermectin provided full protection against heartworm disease [41]. Milbemycin oxime was evaluated at 500 µg/kg when administered 30, 60, 90, and both 60 and 90 days post-inoculation, demonstrating full protection against heartworm disease when administered at 30 days and at both 60 and 90 days post-inoculation [14, 40]. In comparison, moxidectin administered 30 days post-inoculation was 100% efficacious at the lowest dose tested, 1.25 µg/kg [15]. Additionally, moxidectin provided full protection against heartworm disease when administered at 0.5 µg/kg, 2 months post-inoculation, while ivermectin required a ~ 12-fold higher dose and milbemycin oxime, a 1000-fold higher dose for full protection. All of these early evaluations were performed against strains of heartworm that were susceptible to MLs.

Studies evaluating the efficacy of MLs have demonstrated that increasing the dose and number of sequential doses administered increases the efficacy against ML-resistant strains of heartworm. Moxidectin and other MLs have been evaluated for efficacy against the genetically confirmed ML-resistant strains JYD-34, ZoeLA, and ZoeMO. Oral moxidectin administered at 3, 6, 12, and 24 µg/kg demonstrated preventive efficacy of 19%, 25.5%, 33.3%, and 53.2%, respectively, when administered as a single dose 30 days post-inoculation with 50 JYD-34 L3. Additionally, in this same study when oral moxidectin was administered every 30 days for three consecutive months at 3 µg/kg, adult heartworm recovery was reduced by 44.4%, demonstrating an increased efficacy at this dose with repeated monthly administration [48]. Against this same strain, JYD-34, 100% efficacy was achieved when oral moxidectin was administered monthly at 40 µg/kg or 60 µg/kg for three consecutive months, and a 24 µg/kg dose administered monthly for three consecutive months was highly effective (98.8%) (Table 2) [48].

**Table 2:** Preventive efficacy of orally administered moxidectin against macrocyclic lactone-resistant heartworm strains utilizing various dosages and dosage regimens [48]

Study	Strain^a^	Treatment^b^	Dosage (µg/kg)	Days of treatment	Preventive efficacy (%)
1	JYD-34	Placebo	0	0, 30, 60	–
	JYD-34	Moxidectin	3	0	19
	JYD-34	Moxidectin	3	0, 30, 60	44.4
	JYD-34	Moxidectin	6	0	25.5
	JYD-34	Moxidectin	12	0	33.3
	JYD-34	Moxidectin	24	0	53.2
2	JYD-34	Placebo	0	0, 28, 56	–
	JYD-34	Moxidectin	24	0, 28, 56	98.8
	JYD-34	Moxidectin	40	0, 28, 56	100
	JYD-34	Moxidectin	60	0, 28, 56	100
	ZoeLA	Placebo	0	0, 28, 56	–
	ZoeLA	Moxidectin	3	0	44.4
	ZoeLA	Moxidectin	24	0, 28, 56	99.5
	ZoeLA	Moxidectin	40	0, 28, 56	99.5
	ZoeLA	Moxidectin	60	0	88.2
	ZoeLA	Moxidectin	60	0, 28, 56	100
3	ZoeMO	Placebo	0	0, 28, 56	–
	ZoeMO	Moxidectin	3	0	82.1
	ZoeMO	Moxidectin	24	0, 28, 56	99.5
	ZoeMO	Moxidectin	40	0, 28, 56	100
	ZoeMO	Moxidectin	60	0, 28, 56	100

^a^Each dog inoculated with 50 third-stage larvae of the specific strain ~ 1 month prior to the first treatment

^b^For each treatment group, *n* = 5

Oral moxidectin has also been evaluated against the resistant strains ZoeMO and ZoeLA for efficacy in the prevention of heartworm disease. When administered as three consecutive oral monthly dosages, 60 µg/kg moxidectin was 100% effective in preventing the development of both the ZoeLA and ZoeMO resistant strains. Efficacy was reduced to 88.2% when only a single 60 µg/kg oral moxidectin dose was administered against the ZoeLA strain, demonstrating improved efficacy with consecutive monthly administration (Table 2) [48].

The 24 µg/kg moxidectin dose was selected for further development in Simparica Trio based on a number of scientific and commercial considerations [48]. In two additional laboratory studies, the efficacy of 24 µg/kg moxidectin administered orally for either four or six consecutive months was compared directly to Heartgard^®^ Plus (ivermectin/pyrantel, Boehringer Ingelheim Animal Health, Ingelheim, Germany), Interceptor^®^ Plus (milbemycin oxime/praziquantel, Elanco, Greenfield, IN, USA), with both commercial products administered according to the labels, and an untreated control for a total of six consecutive months [49]. Studies used the ML-resistant strains ZoeLA and JYD-34, with each study animal receiving an inoculation of 50 L3 on day −30. A necropsy was performed approximately 9 months post-inoculation (3 months after the last of the six monthly doses) for all study animals in both studies. In the study with the ZoeLA strain, four or six consecutive monthly treatments of 24 µg/kg moxidectin were ≥ 96.1% efficacious, however, six monthly treatments of Heartgard Plus and Interceptor Plus were only 18.7% and 21.2% efficacious, respectively (Table 3) [49]. In the study with JYD-34, four or six consecutive monthly treatments of 24 µg/kg moxidectin were 95.9% and 99.3% effective, and six monthly treatments of Heartgard Plus and Interceptor Plus were 63.9% and 54.6% effective, respectively (Table 4) [49]. These data indicate that the increased dose of oral moxidectin provides greater efficacy against ML-resistant heartworm strains when compared to commercially available oral heartworm preventives and is a viable strategy for the management of ML resistance in the field.

**Table 3:** Preventive efficacy of various orally administered macrocyclic lactones against the macrocyclic lactone-resistant heartworm strain ZoeLA

Study	Treatment	Dosage (µg/kg)	Day of inoculation^a^	Days of treatment	Preventive efficacy (%)	References
1	Placebo	0	−30	0, 30, 60, 90, 120, 150	–	[50]
	Simparica Trio (moxidectin)	Min. 24	−30	0, 30, 60, 90, 120, 150	97.2	[50]
	Heartgard Plus (ivermectin)	Min. 6	−30	0, 30, 60, 90, 120, 150	8.5	[50]
	Interceptor Plus (milbemycin oxime)	Min. 500	−30	0, 30, 60, 90, 120, 150	35.9	[50]
2	Placebo	0	−30	0, 30, 60, 90, 120, 150	–	[49]
	Moxidectin	24	−30	0, 30, 60, 90	96.8	[49]
	Moxidectin	24	−30	0, 30, 60, 90, 120, 150	96.1	[49]
	Heartgard Plus (ivermectin)	Min. 6	−30	0, 30, 60, 90, 120, 150	18.7	[49]
	Interceptor Plus (milbemycin oxime)	Min. 500	−30	0, 30, 60, 90, 120, 150	21.2	[49]

^a^Each dog inoculated with 50 third-stage larvae (L3) of the ZoeLA, macrocyclic lactone-resistant strain

**Table 4:** Preventive efficacy of various macrocyclic lactones against the macrocyclic lactone-resistant heartworm strain JYD-34

Study	Treatment	Route of administration	Dosage (µg/kg)	Day of inoculation^a^	Days of treatment	Preventive efficacy (%)	References
1	Placebo	SC	0	−30	0	–	[54]
	ProHeart 12 (moxidectin)	SC	500	−30	0	100	[54]
	Heartgard Plus (ivermectin)	Oral	Min. 6	−30	0, 30, 60, 90, 120, 150	10.5	[54]
	Interceptor Plus (milbemycin oxime)	Oral	Min. 500	−30	0, 30, 60, 90, 120, 150	14.6	[54]
2	Placebo	SC	0	165	0	–	[54]
	ProHeart 12 (moxidectin)	SC	500	165	0	98.3	[54]
	Heartgard Plus (ivermectin)	Oral	Min. 6	165	0, 30, 60, 90, 120, 150, 180, 210, 240, 270, 300, 330	37.7	[54]
	Interceptor Plus (milbemycin oxime)	Oral	Min. 500	165	0, 30, 60, 90, 120, 150, 180, 210, 240, 270, 300, 330	34.9	[54]
3	Placebo	Oral	0	−30	0, 30, 60, 90, 120, 150	–	[49]
	Moxidectin	Oral	24	−30	0, 30, 60, 90	95.9	[49]
	Moxidectin	Oral	24	−30	0, 30, 60, 90, 120, 150	99.3	[49]
	Heartgard Plus (ivermectin)	Oral	Min. 6	−30	0, 30, 60, 90, 120, 150	63.9	[49]
	Interceptor Plus (milbemycin oxime)	Oral	Min. 500	−30	0, 30, 60, 90, 120, 150	54.6	[49]

^a^Each dog inoculated with 50 third-stage larvae (L3) of the JYD-34, macrocyclic lactone-resistant strain

In a recent laboratory study, Simparica Trio, containing 24 µg/kg moxidectin, 1.2 mg/kg sarolaner and 5 mg/kg pyrantel, and Heartgard Plus and Interceptor Plus (*n* = 6) were administered in six consecutive monthly oral doses at the labeled dose rates after receiving an inoculation of 50 ZoeLA strain *D. immitis* L3 on day −30 [50]. Dogs were necropsied approximately 9 months post-inoculation and evaluated for the presence of circulating MF and heartworm antigen on days 180, 210, and 236. Preventive efficacy of 97.2% in the Simparica Trio-treated group was demonstrated, with all of the treated dogs having ≤ 3 worms present. No dogs treated with Simparica Trio tested positive for the presence of circulating MF at any time during this study, with only two of the six dogs testing positive for heartworm antigen. All six dogs treated with Heartgard Plus and Interceptor Plus had adult worms present at necropsy, with a geometric mean of 32.5 and 22.8 adults recovered, and preventive efficacy of 8.5% and 35.9%, respectively (Table 3) [50]. On day 236, all Heartgard Plus- and Interceptor Plus-treated dogs were positive for the presence of heartworm antigen, and five of the total six dogs for each group were positive for circulating MF, indicating the possibility of infected dogs being able to transmit ML-resistant heartworm strains despite proper use of these preventive products. With very similar results to those obtained in the previous study [49], using 24 µg/kg moxidectin dose as a standalone, these data confirm the robust efficacy of 24 µg/kg moxidectin against the ML-resistant ZoeLA heartworm strain.

The preventive efficacy of Simparica Trio was also evaluated in a multicenter field study, enrolling a total of 410 dogs (365 included in the efficacy evaluation) from 23 different veterinary clinics across the USA. Dogs were administered 11 consecutive monthly doses of either Heartgard Plus (6–12 µg/kg ivermectin and 5–10 mg/kg pyrantel) or Simparica Trio (24–48 µg/kg moxidectin, 2–4 mg/kg sarolaner, and 5–10 mg/kg pyrantel) [51]. All dogs enrolled in the study tested negative for the presence of circulating MF and adult heartworm antigen on days 1/0 (before treatments), 120, and 240. Compliance in administering Heartgard Plus and Simparica Trio in this study was rigorously documented, ensuring that all dogs in efficacy evaluations received their required doses at the appropriate time. Of the 246 dogs that were eligible for efficacy inclusion that received Simparica Trio with complete compliance, no dogs tested positive for MF or heartworm antigen at any point throughout the study, while two dogs of the total 119 eligible for efficacy evaluation that received Heartgard Plus in complete compliance tested positive for heartworm antigen on day 330, with one of these dogs also testing positive for MF. The two dogs treated with Heartgard Plus that became positive for adult heartworms during the study, even with 11 months of documented compliance were from Livonia, Louisiana (LA) in the LMRV [51]. Based on the data presented above, Simparica Trio, containing a minimum of 24 µg/kg moxidectin, will likely provide robust heartworm prevention against the strains to which most dogs in the USA will likely be exposed, including those that may be resistant to MLs.

### Injectable moxidectin

ProHeart^®^ 6 [0.17 mg/kg (170 µg/kg) moxidectin] and ProHeart^®^ 12 [0.5 mg/kg (500 µg/kg) moxidectin] are the only injectable heartworm preventives, and these products offer extended-release moxidectin for the continuous prevention of heartworm disease in dogs for 6 and 12 months, respectively. ProHeart 6 and 12 offer a safe and reliable heartworm preventive option without owners having to comply with monthly administration. Data show that, on average, owners using a monthly product for heartworm prevention administer only half of the prescribed monthly doses necessary to provide complete heartworm prevention [52]. Once ProHeart is administered, compliance is guaranteed to be 100% for the 6- or 12-month dosing interval. In addition to the increased compliance as compared to monthly administered products, data indicate that ProHeart formulations (0.17 mg/kg; ProHeart 6) provide 100% prophylactic activity for one full year against ML-susceptible heartworms, demonstrating the robust, persistent efficacy of the ProHeart formulation against experimentally induced ML-susceptible heartworms [53]. The persistent efficacy observed in ProHeart 6 and 12 is a function of the inherent physicochemical properties of moxidectin, including long half-life and increased lipophilicity and potency, together with the extended-release properties of the formulation [39]. Anaphylactic and anaphylactoid reactions may occur in some dogs following administration of ProHeart 6/12 alone or with vaccines, and they are available through a restricted distribution program to veterinarians that have completed the RiskMAP training and certification module.

The efficacy of ProHeart 12, Heartgard Plus, and Interceptor Plus against the ML-resistant strain JYD-34 was evaluated in two different laboratory studies, in which dogs were either inoculated with 50 *D. immitis* L3 30 or 165 days after initiating treatment, respectively [54]. In the first study, where dogs were inoculated on day −30, dogs (*n* = 6) were either treated with a single dose of ProHeart 12 on day 0 or administered six consecutive monthly oral treatments of Heartgard Plus or Interceptor Plus on days 0, 30, 60, 90, 120, and 150. All dogs were necropsied on day 185, approximately 8 months post-inoculation. The preventive efficacy of ProHeart 12 was 100%, with no dogs having any adult worms at necropsy. In comparison, all six dogs in both the Heartgard Plus- and Interceptor Plus-treated groups, had adult worms present at necropsy, demonstrating an overall preventive efficacy of 10.5% and 14.6% respectively (Table 4) [54]. In the second laboratory study, dogs were inoculated on day 165, in order to compare efficacies against an ML-resistant strain in the middle of the treatment regimen. For this study, dogs (*n* = 6 for each treatment group) were either treated with a single dose of ProHeart 12 on day 0 or administered six consecutive oral monthly doses before and after inoculation with Heartgard Plus or Interceptor Plus, for a total of 12 consecutive monthly treatments [54]. All dogs were necropsied on day 360, approximately 7 months post-inoculation. Adult heartworms were present in all dogs treated with either Heartgard Plus or Interceptor Plus, with preventive efficacies of 37.7% and 34.9% respectively. The preventive efficacy of those dogs treated with ProHeart 12 was 98.3%, with only four of the total six dogs containing a single worm each (Table 4) [54]. These studies indicate that a single injection of ProHeart 12 is highly efficacious (≥ 98%) against a known ML-resistant strain when exposed at both the start and middle of the treatment regimen.

As claims of lack of efficacy for MLs, and confirmed cases of ML resistance continue to increase, field efficacy trials become extremely important in evaluating the efficacy against current natural strains circulating that could be a mixture of susceptible and potentially ML-resistant strains. A field efficacy study evaluated the preventive efficacy of Heartgard Plus and ProHeart 12 with client-owned dogs from 19 veterinary clinics across the USA. Dogs received either 20 consecutive monthly doses of Heartgard Plus or two annual doses of ProHeart 12, with a total of 218 dogs receiving Heartgard Plus and 236 dogs receiving ProHeart 12 [55]. The study was designed to test the efficacy of treatment in dogs over an initial exposure period of 12 months. However, in order to determine if dogs may have been exposed at the end of the 12-month period became infected, all animals needed to be followed for a final antigen test 8 months after the end of the 12-month exposure period. The second ProHeart 12 dose and the additional eight Heartgard Plus doses provided heartworm prevention during this period. Of the 236 dogs that received two annual doses of ProHeart 12, no animals tested positive for the presence of heartworm antigen or circulating MF at any time during this study, indicating complete heartworm prevention for the 12-month evaluation period. Four of the total 218 dogs that received 20 consecutive monthly doses of Heartgard Plus tested positive for the presence of adult heartworm infection during the study. Of these four dogs, three tested positive for heartworm antigen and circulating MF, with the fourth testing positive solely for the presence of heartworm antigen. All four cases of breakthrough infection with Heartgard Plus occurred in the LMRV, with two dogs from Zachary, LA, one dog from Lake Charles, LA, and one dog from Memphis, Tennessee. Compliance for administration of Heartgard Plus in the study was rigorously documented, ensuring that all dogs received their required doses at the appropriate time. Three of the four Heartgard Plus-treated dogs were also positive for MF, even with continued Heartgard Plus treatment, indicating that these treatment failures may have been due to exposure to ML-resistant heartworms. There was a significant difference between the ProHeart 12 and Heartgard Plus groups, indicating that ProHeart 12 performed better than Heartgard Plus in preventing heartworm disease in this field study, confirming the robust efficacy of ProHeart 12 under field conditions [55]. The difference in the performance of these two products in this study is likely related to the inherent properties of moxidectin and the dose of moxidectin in the product, in addition to the continuous presence of the active compound through the dosing period made possible by the ProHeart 12 extended-release formulation.

The transmission of heartworm disease is dependent on the ingestion of MF by female mosquitoes from a patent host. Therefore, the reduction and elimination of circulating MF in infected animals is important in reducing the transmission and prevalence of this disease. Topical moxidectin (Advantage^®^ Multi, Bayer Animal Health, Shawnee Mission, KS, USA) is the only ML FDA-approved for the elimination of circulating MF in canine heartworm disease, with a > 99% reduction in MF observed compared to untreated controls 7 days post-treatment [56]. The 0.5 mg/kg moxidectin dose in ProHeart 12 is also highly microfilaricidal, with > 97% reduction in MF reported 7 days post-treatment [57]. The results for both Advantage Multi and ProHeart 12 are for strains susceptible to MLs. Evaluation of ProHeart 6 and ProHeart 12 in the reduction of circulating MF against an ML-resistant strain, ZoeMO, demonstrated a > 90% reduction in MF by 28 and 42 days post-treatment respectively, and > 96% on day 84, after a single subcutaneous dose [58]. Based on these data, injectable moxidectin may offer the potential for microfilarial reduction, even against ML-resistant strains providing additional opportunity to reduce the transmission of heartworm.

### Topical moxidectin

Moxidectin can be administered topically for the prevention of heartworm disease and is available for dogs, cats, and ferrets (Advantage Multi^®^; Coraxis^®^, Bayer Animal Health). The increased dose of moxidectin administered in Advantage Multi/Coraxis, 2.5 mg/kg (2500 µg/kg), provides efficacy for the treatment and control of gastrointestinal nematodes, including hookworm, roundworm, and whipworm species in dogs.

Topical moxidectin is the only FDA-approved treatment for circulating MF in heartworm-positive dogs [56, 59, 60]. In two laboratory studies, naturally infected dogs and dogs experimentally infected by surgical transplantation of adult worms were administered topical Advantage Multi on days 0 and 28, with blood samples collected at various times post-treatment. Fourteen days post-treatment, dogs in both studies had a > 99% reduction in circulating MF observed compared to the untreated control groups. This reduction of > 99% persisted through day 42, the study end, with one dog still testing positive for the presence of circulating MF in the naturally infected dogs and a geometric mean of 7.1 MF/ml in the experimentally infected dogs on day 42 [56, 60].

Due to the high dose of moxidectin administered in Advantage Multi/Coraxis, accumulation occurs with subsequent monthly doses, with a steady state of mean serum concentration reached after four monthly topical applications [61]. This steady state of moxidectin is fully efficacious in the prevention of heartworm disease, as dogs inoculated with 50 susceptible *D. immitis* L3 28 days after four monthly topical treatments had no adult heartworms recovered at necropsy [61]. The mean terminal phase half-life for the topical administration of moxidectin is 28 days, therefore providing an extended duration of time over the mean efficacious dose (MED) as compared to that of oral ivermectin [61]. Advantage Multi has also demonstrated efficacy against ML-resistant heartworms, with 100% preventive efficacy against the JYD-34 strain with a single dose administered 30 days post-inoculation with *D. immitis* L3 [30]. There was a report of < 100% efficacy in a second study using a single dose of Advantage Multi that was not published (McCall, unpublished data).

Despite the benefits of topical moxidectin for the prevention of heartworm disease, there are disadvantages to this topical administration route. Improper owner administration of topical products can lead to inadequate exposure and absorption of the active ingredients, and subsequently sub-efficacious doses to the dog. Additionally, topical products can lead to increased environmental contamination due to exposure of household items and other members of the household to the product. Also important is that accidental oral administration of ≥ 40% of the topical dose (total of 1 mg/kg dose) to a dog with the MDR-1 defect can result in severe adverse effects, resulting in a black box warning on the label.

## Conclusions

Resistance of some strains of *D. immitis* to MLs has been confirmed in the USA. Despite continued research and investigation, products containing novel compounds for the successful prevention of heartworm disease have not been brought to market. Therefore, currently the management of ML resistance is likely best achieved through the use of optimized formulations of MLs that focus on increased dose rate, and selection of MLs with optimal physicochemical and pharmacokinetic properties. Moxidectin is one such ML with favorable attributes, such as high potency against heartworms, long half-life, and greater margin of safety, that make it ideal for optimization in various formulations. With the continued and increasing threat of ML resistance in the USA, veterinarians play a critical role in educating clients on the importance of proper heartworm preventive administration to protect their pets from all strains of heartworms to which their pets are potentially exposed, especially considering the potential for the further spread of ML-resistant heartworm strains in the USA.

## Data Availability

The datasets used and/or analyzed during the current study are available from the corresponding author on reasonable request.

## Abbreviations

APIs: Active pharmaceutical ingredients
ES: Excretory-secretory
FDA: Food and Drug Administration
GABA_A_: Gamma-aminobutyric acid type A-gated chloride
L3: Infective third-stage larvae
L4: Fourth-stage larvae
L5: Juvenile adult worms
LA: Louisiana
LMRV: Lower Mississippi River Valley
MLs: Macrocyclic lactones
MED: Mean efficacious dose
MDR-1: Multidrug resistance-1
MF: Microfilariae
P-gps: P-glycoproteins
PDGF: Platelet-derived growth factor

## References

[1] Kume S, Itagaki S (1955). On the life-cycle of *Dirofilaria immitis* in the dog as the final host. Br Vet J.

[2] Kotani T, Powers KG (1982). Developmental stages of *Dirofilaria immitis* in the dog. Am J Vet Res.

[3] Orihel TC (1961). Morphology of the larval stages of *Dirofilaria immitis* in the dog. J Parasitol.

[4] Rawlings CA, Losonsky JM, Lewis RE, McCall JW (1981). Development and resolution of radiographic lesions in canine heartworm disease. J Am Vet Med Assoc.

[5] Rawlings CA (1982). Clinical laboratory evaluations of seven heartworm infected beagles: during disease development and following treatment. Cornell Vet.

[6] Schaub RG, Rawlings CA, Keith JC (1981). Platelet adhesion and myointimal proliferation in canine pulmonary arteries. Am J Pathol.

[7] Schaub RG, Rawlings CA, Keith JC (1981). Effect of long-term aspirin treatment on platelet adhesion to chronically damaged canine pulmonary arteries. Thromb Haemost.

[8] Rawlings CA, Pendersen D, Folcher MA (1986). The pulmonary response to adult *Dirofilaria immitis*. Heartworm disease in dogs and cats.

[9] McCracken MD, Patton S (1993). Pulmonary arterial changes in feline dirofilariasis. Vet Pathol.

[10] Ames MK, Atkins CE (2020). Treatment of dogs with severe heartworm disease. Vet Parasitol..

[11] Bowman DD (2012). Heartworms, macrocyclic lactones, and the specter of resistance to prevention in the United States. Parasit Vectors.

[12] McCall JW (2005). The safety-net story about macrocyclic lactone heartworm preventives: a review, an update, and recommendations. Vet Parasitol.

[13] Campbell WC, Campbell WC (1989). Use of ivermectin in dogs and cats. Ivermectin and abamectin.

[14] Grieve RB, Frank GR, Stewart VA, Parsons JC, Belasco DL, Hepler DI (1991). Chemoprophylactic effects of milbemycin oxime against larvae of *Dirofilaria immitis* during prepatent development. Am J Vet Res.

[15] McTier T, McCall JW, Dzimianski MT, Aguilar R, Wood I. Prevention of experimental heartworm infection in dogs with single oral doses of moxidectin. In: proceedings of the heartworm symposium 1992; Austin, TX: American Heartworm Society: 165–8.

[16] McTier TL, Shanks DJ, Watson P, McCall JW, Genchi C, Six RH (2000). Prevention of experimentally induced heartworm (*Dirofilaria immitis*) infections in dogs and cats with a single topical application of selamectin. Vet Parasitol.

[17] Lok JB KD, McCall JW, Dzimianski MT, Cleale RM, Wang GT, et al. Six-month prophylactic efficacy of an injectable, sustained-release formulation of moxidectin against *Dirofilaria immitis* infection: a two-center study. In: recent advances in heartworm disease symposium 2001; San Antonio, TX: American Heartworm Society: 149–57.

[18] Arther RG, Bowman DD, Slone RL, Travis LE (2005). Imidacloprid plus moxidectin topical solution for the prevention of heartworm disease (*Dirofilaria immitis*) in dogs. Parasitol Res.

[19] Lok JB, Knight DH, Wang GT, Doscher ME, Nolan TJ, Hendrick MJ (2001). Activity of an injectable, sustained-release formulation of moxidectin administered prophylactically to mixed-breed dogs to prevent infection with *Dirofilaria immitis*. Am J Vet Res.

[20] Wolstenholme AJ, Maclean MJ, Coates R, McCoy CJ, Reaves BJ (2016). How do the macrocyclic lactones kill filarial nematode larvae?. Invert Neurosci.

[21] Geary TG, Moreno Y (2012). Macrocyclic lactone anthelmintics: spectrum of activity and mechanism of action. Curr Pharm Biotechnol.

[22] Wolstenholme AJ, Rogers AT (2005). Glutamate-gated chloride channels and the mode of action of the avermectin/milbemycin anthelmintics. Parasitology.

[23] Geyer J, Janko C (2012). Treatment of MDR1 mutant dogs with macrocyclic lactones. Curr Pharm Biotechnol.

[24] Harischandra H, Yuan W, Loghry HJ, Zamanian M, Kimber MJ (2018). Profiling extracellular vesicle release by the filarial nematode *Brugia malayi* reveals sex-specific differences in cargo and a sensitivity to ivermectin. PLoS Negl Trop Dis..

[25] Moreno Y, Nabhan JF, Solomon J, Mackenzie CD, Geary TG (2010). Ivermectin disrupts the function of the excretory-secretory apparatus in microfilariae of *Brugia malayi*. Proc Natl Acad Sci USA.

[26] Vatta AF, Dzimianski M, Storey BE, Camus MS, Moorhead AR, Kaplan RM (2014). Ivermectin-dependent attachment of neutrophils and peripheral blood mononuclear cells *to Dirofilaria immitis* microfilariae *in vitro*. Vet Parasitol.

[27] Rehm CJ. AHS survey finds increase in heartworm cases. 2017. https://www.heartwormsociety.org/veterinary-resources/veterinary-education/ahs-board-speaks-out/368-ahs-survey-finds-increase-in-heartworm-cases. Accessed 08 Oct 2021.

[28] Bourguinat C, Lee ACY, Lizundia R, Blagburn BL, Liotta JL, Kraus MS (2015). Macrocyclic lactone resistance in *Dirofilaria immitis*: Failure of heartworm preventives and investigation of genetic markers for resistance. Vet Parasitol.

[29] Pulaski CN, Malone JB, Bourguinat C, Prichard R, Geary T, Ward D (2014). Establishment of macrocyclic lactone resistant *Dirofilaria immitis* isolates in experimentally infected laboratory dogs. Parasit Vectors.

[30] Blagburn BL, Arther RG, Dillon AR, Butler JM, Bowles JV, von Simson C (2016). Efficacy of four commercially available heartworm preventive products against the JYD-34 laboratory strain of *Dirofilaria immitis*. Parasit Vectors.

[31] Ballesteros C, Pulaski CN, Bourguinat C, Keller K, Prichard RK, Geary TG (2018). Clinical validation of molecular markers of macrocyclic lactone resistance in *Dirofilaria immitis*. Int J Parasitol Drugs Drug Resist.

[32] Maclean MJ, Savadelis MD, Coates R, Dzimianski MT, Jones C, Benbow C (2017). Does evaluation of *in vitro* microfilarial motility reflect the resistance status of *Dirofilaria immitis* isolates to macrocyclic lactones. Parasit Vectors.

[33] McTier TL, Six RH, Pullins A, Chapin S, McCall JW, Rugg D (2017). Efficacy of oral moxidectin against susceptible and resistant isolates of *Dirofilaria immitis* in dogs. Parasit Vectors.

[34] Prichard RK, Geary TG (2019). Perspectives on the utility of moxidectin for the control of parasitic nematodes in the face of developing anthelmintic resistance. Int J Parasitol Drugs Drug Resist.

[35] Ledesma N, Harrington L (2011). Mosquito vectors of dog heartworm in the United States: vector status and factors influencing transmission efficiency. Top Companion Anim Med.

[36] Bourguinat C, Keller K, Bhan A, Peregrine A, Geary T, Prichard R (2011). Macrocyclic lactone resistance in *Dirofilaria immitis*. Vet Parasitol.

[37] Drake J, Parrish RS (2019). Dog importation and changes in heartworm prevalence in Colorado 2013–2017. Parasit Vectors.

[38] Prichard R, Menez C, Lespine A (2012). Moxidectin and the avermectins: consanguinity but not identity. Int J Parasitol Drugs Drug Resist.

[39] Al-Azzam SI, Fleckenstein L, Cheng KJ, Dzimianski MT, McCall JW (2007). Comparison of the pharmacokinetics of moxidectin and ivermectin after oral administration to beagle dogs. Biopharm Drug Dispos.

[40] Grieve RB, Frank GR, Stewart VA, Parsons JC, Abraham D, MacWilliams PS, Hepler D. Effect of dosage and dose timing on heartworm (*Dirofilaria immitis*) chemoprophylaxis with milbemycin. In: proceedings of the American heartworm symposium 1989; Washington, DC: American Heartworm Society; 1989. pp. 121–4.

[41] Paul AJ, Todd KS, Acre KE, Plue RE, Wallace DH, French RA (1991). Efficacy of ivermectin chewable tablets and two new ivermectin tablet formulations against *Dirofilaria immitis* larvae in dogs. Am J Vet Res.

[42] Paul AJ, Todd KS, Sundberg JP, DiPietro JA, McCall JW (1986). Efficacy of ivermectin against *Dirofilaria immitis* larvae in dogs 30 and 45 days after induced infection. Am J Vet Res.

[43] Menez C, Sutra JF, Prichard R, Lespine A (2012). Relative neurotoxicity of ivermectin and moxidectin in MDR1ab (−/−) mice and effects on mammalian GABA(A) channel activity. PLoS Negl Trop Dis.

[44] Geyer J, Klintzsch S, Meerkamp K, Wohlke A, Distl O, Moritz A (2007). Detection of the nt230(del4) MDR1 mutation in white Swiss shepherd dogs: case reports of doramectin toxicosis, breed predisposition, and microsatellite analysis. J Vet Pharmacol Ther.

[45] Paul AJ, Tranquilli WJ, Seward RL, Todd KS, DiPietro JA (1987). Clinical observations in collies given ivermectin orally. Am J Vet Res.

[46] Geyer J, Doring B, Godoy JR, Moritz A, Petzinger E (2005). Development of a PCR-based diagnostic test detecting a nt230(del4) MDR1 mutation in dogs: Verification in a moxidectin-sensitive Australian shepherd. J Vet Pharmacol Ther.

[47] Paul AJ, Tranquilli WJ, Hutchens DE (2000). Safety of moxidectin in avermectin-sensitive collies. Am J Vet Res.

[48] McTier TL, Six RH, Pullins A, Chapin S, Kryda K, Mahabir SP (2019). Preventive efficacy of oral moxidectin at various doses and dosage regimens against macrocyclic lactone-resistant heartworm (*Dirofilaria immitis*) strains in dogs. Parasit Vectors.

[49] Kryda K, Holzmer SJ, Everett WR, McCall JW, Mahabir SP, McTier TL (2020). Preventive efficacy of four or six monthly oral doses of 24 µg/kg moxidectin compared to six monthly doses of Heartgard^®^ Plus or Interceptor^®^ Plus against macrocyclic lactone-resistant heartworm (*Dirofilaria immitis*) strains in dogs. Parasit Vectors.

[50] Myers JAE, Holzmer S, McCall JW, Mahabir SP, McTier TL, Maeder SJ (2021). Preventive efficacy of six monthly oral doses of Simparica Trio^®^, Heartgard^®^ Plus and Interceptor^®^ Plus against a macrocyclic lactone-resistant strain (ZoeLA) of heartworm (*Dirofilaria immitis*) in dogs. Parasit Vectors.

[51] Kryda K, Six RH, Walsh KF, Holzmer SJ, Chapin S, Mahabir SP (2019). Laboratory and field studies to investigate the efficacy of a novel, orally administered combination product containing moxidectin, sarolaner and pyrantel for the prevention of heartworm disease (*Dirofilaria immitis*) in dogs. Parasit Vectors.

[52] American Animal Hospital Association. Compliance: taking quality care to the next level. A report of the 2009 AAHA compliance follow-up study. Colorado: AAHA; 2009.

[53] Lok JB, Knight DH, Nolan TJ, Grubbs ST, Cleale RM, Heaney K (2005). Efficacy of an injectable, sustained-release formulation of moxidectin in preventing experimental heartworm infection in mongrel dogs challenged 12 months after administration. Vet Parasitol.

[54] McTier TL, Holzmer S, Kryda K, Mahabir S, McCall JW, Trombley J (2021). Comparative preventive efficacy of ProHeart^®^ 12, Heartgard^®^ Plus and Interceptor^®^ Plus against a macrocyclic lactone-resistant strain (JYD-34) of heartworm (*Dirofilaria immitis*) in dogs. Parasit Vectors.

[55] McTier TL, Kryda K, Wachowski M, Mahabir S, Ramsey D, Rugg D (2019). ProHeart® 12, a moxidectin extended-release injectable formulation for prevention of heartworm (*Dirofilaria immitis*) disease in dogs in the USA for 12 months. Parasit Vectors.

[56] Bowman DD, Charles SD, Arther RG, Settje T (2015). Laboratory evaluation of the efficacy of 10% imidacloprid + 25% moxidectin topical solution (Advantage^®^ Multi, Advocate^®^) for the treatment of Dirofilaria immitis circulating microfilariae in dogs. Parasitol Res.

[57] Blagburn BL, Paul AJ, Newton JC, Hutchens DE, Butler JM, Vaughan JL, et al. Safety of moxidectin canine sr (sustained release) injectable in ivermectin-sensitive collies and in naturally infected mongrel dogs. In: recent advances in heartworm disease symposium 2001; San Antonio, TX*:* American Heartworm Society; 2001. pp. 159–163.

[58] McTier TL, Pullins A, Inskeep GA, Gagnon G, Fan H, Schoell A (2017). Microfilarial reduction following ProHeart^®^ 6 and ProHeart^®^ SR-12 treatment in dogs experimentally inoculated with a resistant isolate of *Dirofilaria immitis*. Parasit Vectors.

[59] di Regalbono AF, Di Cesare A, Traversa D, Simonato G, Poser H, Danesi P (2016). Microfilaricidal efficacy of a single administration of Advocate^®^ (Bayer Animal Health) in dogs naturally infected with *Dirofilaria immitis* or *Dirofilaria repens*. Vet Parasitol.

[60] McCall JW, Arther R, Davis W, Settje T (2014). Safety and efficacy of 10% imidacloprid +2.5% moxidectin for the treatment of *Dirofilaria immitis* circulating microfilariae in experimentally infected dogs. Vet Parasitol..

[61] Bowman DD, Grazette AR, Basel C, Wang Y, Hostetler JA (2016). Protection of dogs against canine heartworm infection 28 days after four monthly treatments with Advantage Multi^®^ for dogs. Parasit Vectors.

